# Ultrasonography-Guided Flexor Hallucis Longus Tenotomy for Checkrein Deformity of the Hallux

**DOI:** 10.1016/j.eats.2025.103748

**Published:** 2025-07-16

**Authors:** Akinobu Nishimura, Shigeto Nakazora, Yoshiyuki Senga, Naoya Ito, Aki Fukuda, Ko Kato, Masahiro Hasegawa

**Affiliations:** a Department of Orthopaedic Surgery, Mie University Graduate School of Medicine, Tsu, Japan; b Department of Orthopaedic and Sports Medicine, Mie University Graduate School of Medicine, Tsu, Japan; c Department of Orthopaedic Surgery, Suzuka Kaisei Hospital, Suzuka, Japan

## Abstract

The checkrein deformity of the flexor hallucis longus (FHL)—resulting from tibial, fibular, and/or talar fractures, removal of a fibular graft, and so on—increases with dorsiflexion of the ankle, but plantar flexion of the ankle can partially correct the deformity. In this article, ultrasonography-guided FHL tenotomy for checkrein deformity of the hallux is described. The operative procedure is performed in the supine position, and the ultrasonic probe is placed on the posterior side of the medial malleolus. Then, 1% lidocaine is injected to separate the neurovascular bundle from the FHL under ultrasonography so that the FHL tendon can be picked up safely. The FHL is subsequently cut under direct vision. Weight-bearing and range of motion are allowed as tolerated, according to pain, starting 1 day after surgery. Ultrasonography-guided FHL tenotomy for checkrein deformity of the hallux requires some ultrasound training, but it is a very promising procedure because it is safe and less invasive than open procedures.

Checkrein deformities are rare and involve entrapment or fixed tethering of the flexor hallucis longus (FHL) tendon in the posterior foot, proximal to the flexor retinaculum of the ankle.^1^^,^^2^ This deformity increases with dorsiflexion of the ankle, and plantar flexion of the ankle can partially correct the deformity (Fig 1).^3^ According to previous reports, this deformity results from fibular, calcaneal, and talar fractures; removal of fibular grafts; or contusion soft-tissue injuries of the leg.^1^^,^^2^^,^^4^^,^^5^ In the past, surgical treatment of this deformity was achieved by releasing the FHL tendon from the adhesion site, such as the fracture site, and/or FHL tendon lengthening.^6^ However, if the FHL muscle shows severe atrophic change or patients desire an early return to work or sports, FHL tenotomy is a viable option.

**Fig 1:**
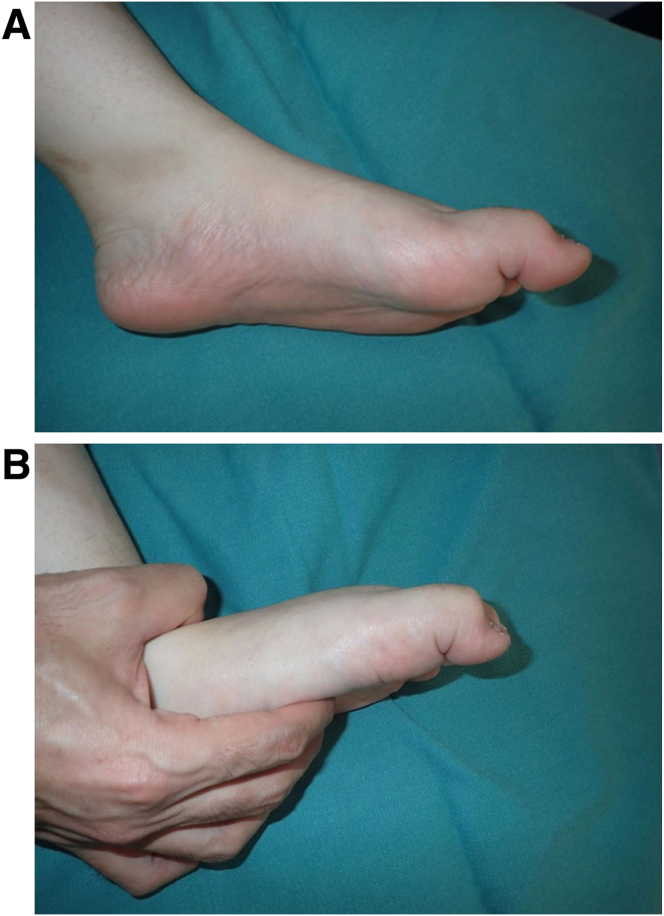
Macroscopic findings of checkrein deformity. (A) With the ankle in plantar flexion, the deformity of the hallux is partially corrected. (B) With the ankle in dorsiflexion, flexion deformity of the hallux becomes more apparent. The patient is in the supine position with the left foot viewed from the medial side.

In this report, an ultrasonography-guided FHL tenotomy technique for checkrein deformity of the hallux is presented. In addition, the usefulness and safety of this procedure are discussed.

## Surgical Technique

A detailed description of the ultrasound technique is provided in Video 1.

### Indications and Contraindications

The described technique is indicated for checkrein deformity of the hallux. This deformity presents as a claw- or hammer-toe deformity. This deformity increases with dorsiflexion of the ankle, and plantar flexion of the ankle can partially correct the deformity. Claw- and hammer-toe deformities are difficult to correct even when the ankle is placed passively in the plantar position. In these cases, joint contracture develops, and this procedure is then contraindicated. A severe atrophic or fibrotic FHL muscle belly is a good indication for this procedure; the patient’s desire to return to sports or work early is a relative indication.

### Ultrasonography-Guided FHL Tenotomy

**Fig 2:**
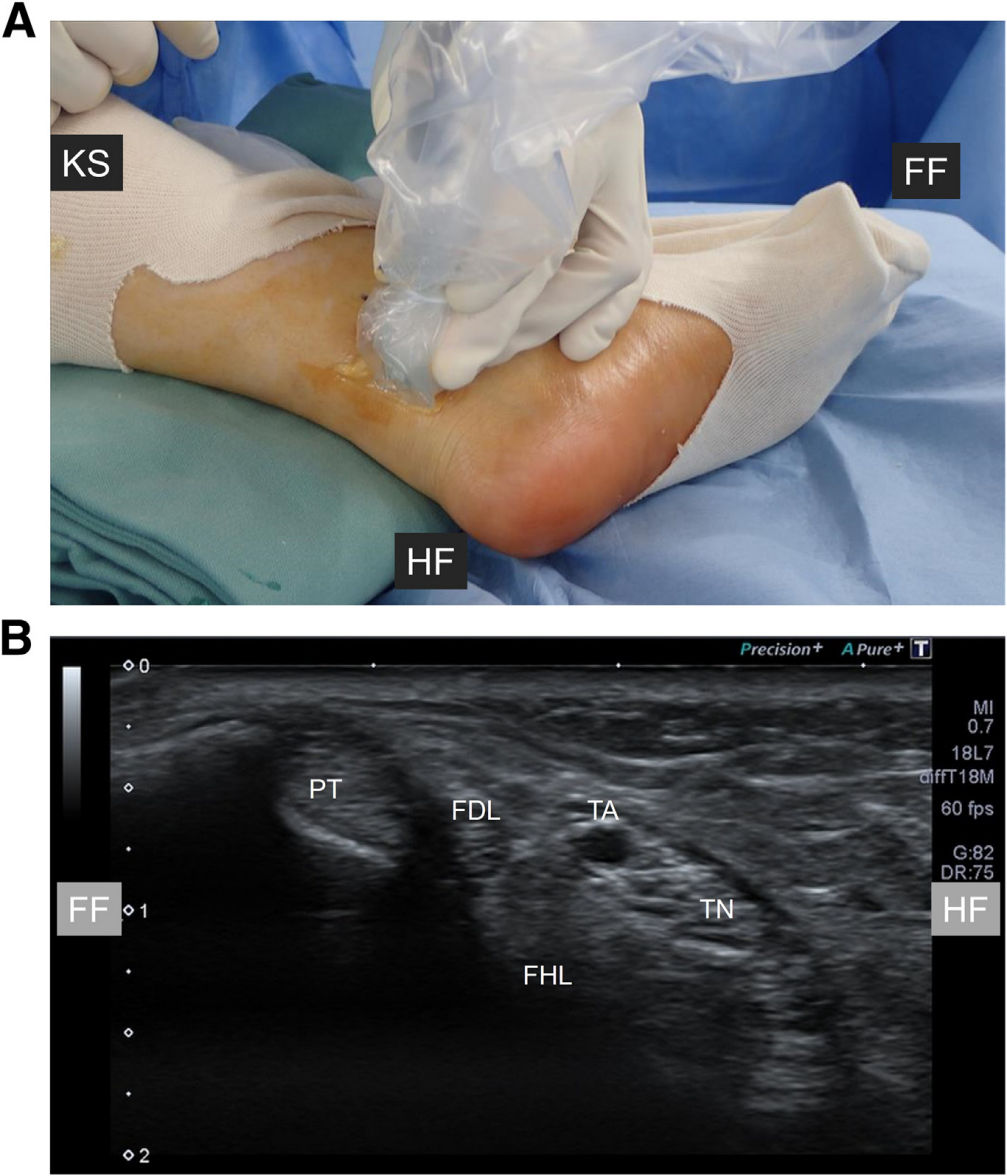
Preoperative setup and ultrasonographic anatomy of medial ankle. (A) The patient is placed in the supine position with the operative leg in the figure-of-4 position. A high-frequency linear ultrasound probe is placed posterior to the medial malleolus of the left ankle. (B) Short-axis ultrasonographic image identifying key anatomic structures. (FDL, flexor digitorum longus tendon; FF, forefoot side; FHL, flexor hallucis longus tendon; HF, hindfoot side; KS, knee side; PT, posterior tibial tendon; TA, tibial artery; TN, tibial nerve.)

**Fig 3:**
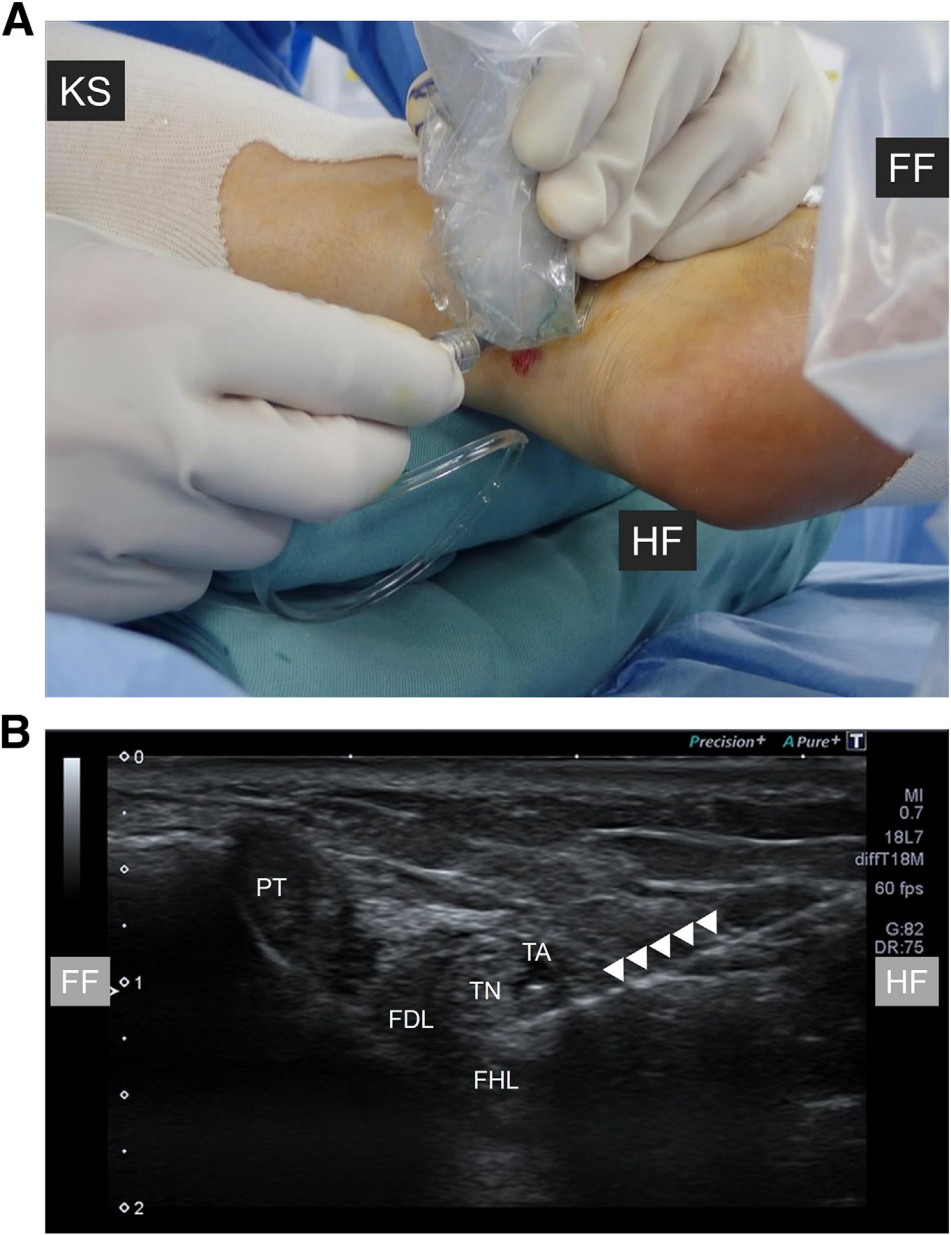
Injection of 1% lidocaine into the left foot to separate neurovascular bundle from flexor hallucis longus (FHL) tendon under ultrasonographic guidance. (A) The needle is inserted from the medial side of the Achilles tendon. The patient remains in the supine figure-of-4 position. (B) An ultrasonographic image shows the needle tip (arrowheads) located between the tibial artery (TA), tibial nerve (TN), and FHL tendon, confirming a safe injection space. (FDL, flexor digitorum longus tendon; FF, forefoot side; HF, hindfoot side; KS, knee side; PT, posterior tibial tendon.)

**Fig 4:**
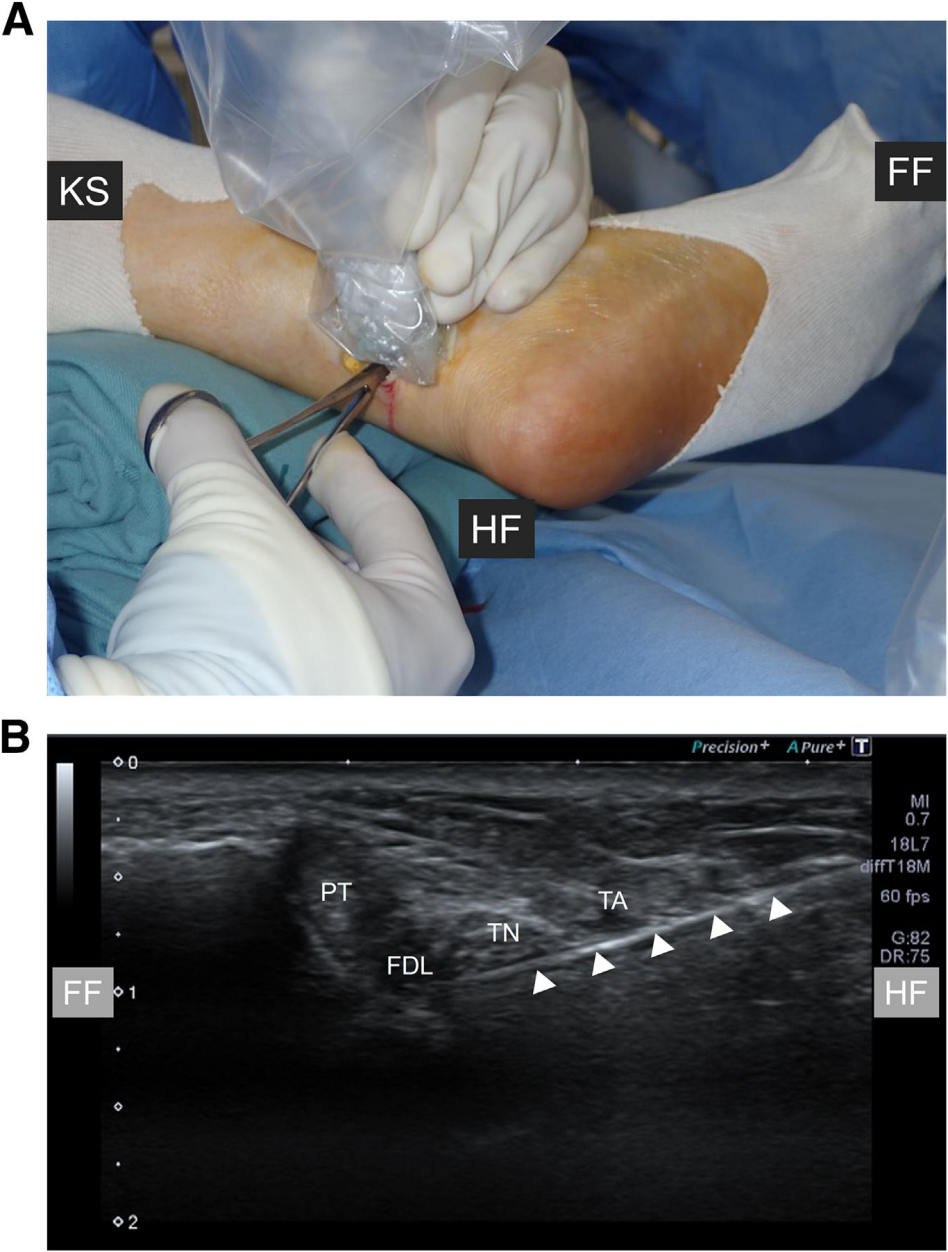
Blunt dissection of the left foot using mosquito Pean forceps under ultrasonographic guidance. (A) A mosquito Pean forceps is inserted from a medial approach while the patient remains supine. (B) The instrument (arrowheads) is shown clearly separating the neurovascular bundle and flexor hallucis longus tendon under ultrasound. (FDL, flexor digitorum longus tendon; FF, forefoot side; HF, hindfoot side; KS, knee side; PT, posterior tibial tendon; TA, tibial artery; TN, tibial nerve.)

**Fig 5:**
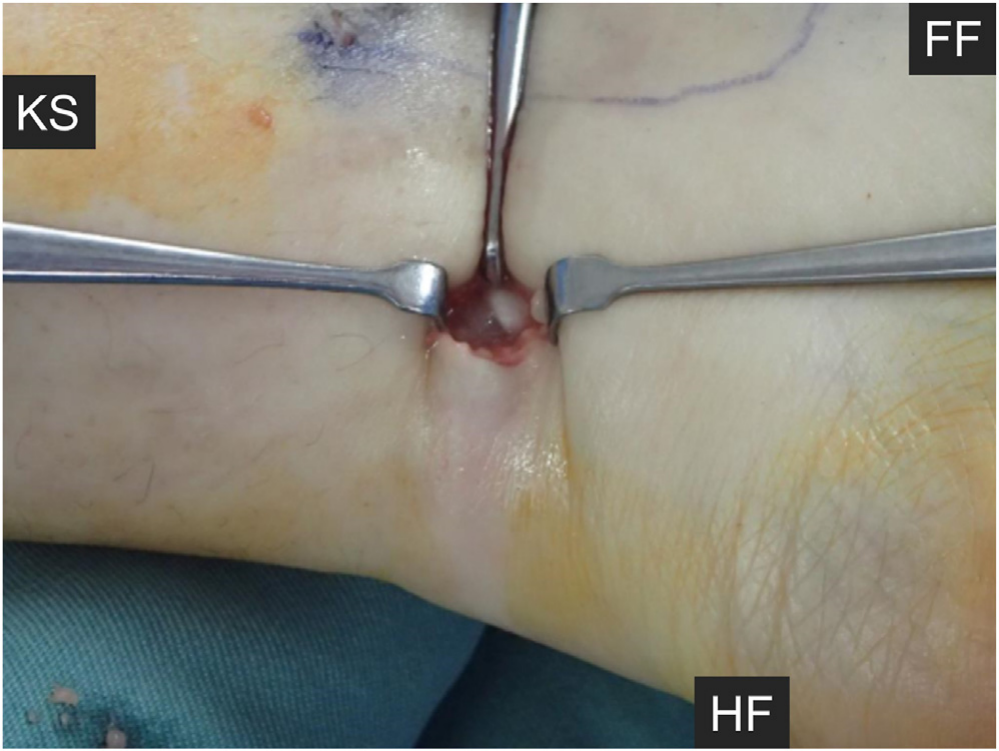
Exposure of flexor hallucis longus tendon of the left foot under direct vision. The tendon is hooked using a blunt instrument after blunt dissection under ultrasonography and then exteriorized through the skin incision for tenotomy. (FF, forefoot side; HF, hindfoot side; KS, knee side.)

Surgery is performed with the patient under general, lumbar spinal, or nerve block anesthesia. The patient is placed in the supine position, and the operative leg is placed in the figure-of-4 position (hip abduction and external rotation with knee flexion). A tourniquet is applied on the thigh for general and lumbar spinal anesthesia or on the proximal lower leg for nerve block anesthesia. An ultrasound machine (Noblus [Hitachi Aloka, Tokyo, Japan], Aplio 300 [Toshiba, Tokyo, Japan], or Snible [Konica Minolta, Tokyo, Japan]) and a high-frequency linear probe are prepared with a sterile ultrasound probe cover and sterile gel (Fuji Medical, Tokyo, Japan). Before skin incision, the positions of the neurovascular bundle (NVB) and FHL are confirmed using ultrasonography (Fig 2). When Doppler ultrasonography is used, the NVB can be easily detected. A 22-gauge needle is inserted between the NVB and FHL, and 1% lidocaine (about 5 mL) is injected to separate the NVB and FHL (Fig 3). A skin incision is made 1 fingerbreadth proximal to the medial malleolus, and blunt dissection between the NVB and FHL is performed with a mosquito Pean forceps (Fig 4). The FHL is isolated with a blunt hook under ultrasonography and exteriorized through the skin incision (Fig 5). Then, the FHL is cut under direct vision. After performing the FHL tenotomy, the surgeon manually assesses whether the great toe can be extended with the ankle in a dorsiflexed position to confirm the improvement of the checkrein deformity. After irrigation, the skin is closed with No. 4-0 Prolene suture (Johnson & Johnson, Tokyo, Japan). Table 1 presents pearls and pitfalls for each step of the procedure.

**Table 1:** Surgical Steps, Pearls, and Pitfalls

Surgical Steps	Pearls	Pitfalls
Observation under ultrasonography	Using Doppler ultrasonography facilitates detection of the NVB. Passive movement of the hallux assists in detecting the FHL tendon.	The FDL tendon can be moved by passive movement of the FHL tendon. The surgeon must not misidentify the FDL as the FHL.
Lidocaine injection to separate NVB and FHL	An ultrasound short-axis image is produced in which the FHL and NVB are simultaneously visible, and a needle is inserted from the medial side of the Achilles tendon. The surgeon should capture the needle in the long axis and inject 1% lidocaine between the FHL and NVB so as not to damage the NVB.	If the ultrasound probe and the injection needle are misaligned, it is difficult to accurately determine the location of the needle tip, so an image is drawn in which the needle is clearly visible.
Skin incision	A skin incision is made medial to the Achilles tendon at the extension of the ultrasound probe where the FHL and NVB are delineated in the short-axis image. The skin incision is made 1 fingerbreadth proximal to the medial malleolus. This is usually slightly more proximal than the location of the medial portal used in ankle hindfoot endoscopy.	If the incision is too close to the Achilles tendon, postoperative symptoms associated with adhesion to the Achilles tendon may occur. The skin incision should not be too distal because the FHL changes direction anteriorly under the sustentaculum tali when the skin incision is distal, resulting in a greater distance between the skin incision and the FHL.
Blunt dissection between NVB and FHL	A short-axis image is produced in which the FHL and NVB are visible in the same field, extending from the skin incision, and a mosquito Pean forceps is placed within the image.	If the ultrasound probe and the mosquito Pean forceps are misaligned, the long-axis image of the mosquito Pean forceps should be firmly delineated because it is impossible to know where the tip of the mosquito device is.
Hooking of FHL tendon with blunt hook	Because the FHL is shortened, it is easier to guide the FHL tendon under direct vision if the hook is placed over the FHL and then the ankle joint and great toe are plantar flexed.	The presence of air in the dissected area makes it difficult to observe the same area by ultrasound.
Cutting of FHL tendon under direct vision	Because the FHL is shortened, the surgical assistant can plantar flex the ankle joint and flex the great toe to give the FHL more room between the tendon and the skin. If, after the FHL is cut, the patient is able to perform passive extension of the great toe with the foot in a dorsiflexed position, this confirms that the checkrein deformity has been improved.	The surgeon should take care not to cut the skin when separating the FHL tendon. The tendon is often compressed by the skin.

FDL, flexor digitorum longus; FHL, flexor hallucis longus; NVB, neurovascular bundle.

Regarding postoperative care, no immobilization is required if the patient undergoes FHL tenotomy alone. Weight-bearing is allowed as tolerated, according to pain, starting 1 day after surgery. Range-of-motion training is also allowed starting 1 day after surgery. If the patient desires a greater level of physical activity, jogging is allowed after suture removal.

## Discussion

The checkrein deformity is a dynamic flexion deformity of the FHL tendon and sometimes results from a tibial fracture. The FHL muscle easily becomes tethered to the healed site of the tibial fracture because the muscle belly of the FHL is larger than that of the other flexors.^6^ Since Clawson^4^ first described claw-toe deformity after fractures of the tibia in 1974, it has been reported in other situations.^1^, ^2^, ^3^^,^^5^^,^^7^ Even in fractures not involving the tibia, the FHL tendon was found to be adherent at the site of the healed fractures.^8^ We have performed the described technique in two cases that resulted from fractures, one case that resulted from the removal of a fibular graft, one case that resulted from compartment syndrome associated with sepsis, and one case that resulted from motor neuron dysfunction because of scleroderma. The patient with scleroderma showed muscle fatty infiltration and muscle fibrosis of the flexor muscles; thus, this patient was thought to show the checkrein deformity, similarly to the post-trauma patients.

The surgical management of checkrein deformity generally consists of release of adhesions at the fracture site and lengthening of the FHL.^8^ However, these procedures usually require long skin incisions, and there is some possibility of recurrence. Female patients may prefer to avoid a long skin incision because of cosmetic concerns. Furthermore, skin problems occur more easily in patients with scleroderma than in those without scleroderma. Therefore, this procedure is suitable for the aforementioned patient with scleroderma. In terms of recurrence, Lee et al.^6^ reported 1 complete recurrence and 2 partial recurrences among their 11 cases. We have observed no recurrences after the present procedure because FHL function is sacrificed due to tenotomy.

The FHL tendon is usually interconnected with the flexor digitorum longus (FDL) tendon.^9^ Because of this, deformities of the lesser toes sometimes coexist with hallux deformity. This interconnection exists from the heel to the first metatarsophalangeal joint on the plantar side of the foot. If the FHL tendon is cut at the metatarsophalangeal joint level, deformities of the lesser toes remain. Therefore, we cut the FHL tendon at the ankle level. Moreover, after the FHL tendon is cut at the ankle level, the flexion power of the FDL is transmitted to the hallux via the interconnections. For the aforementioned reasons, patients do not experience loss of active plantar flexion of the hallux, and hallux flexion power does not decrease more than expected.

FHL tenotomy can be performed using three approaches: open, endoscopic, and ultrasonography guided. Each procedure has its advantages and disadvantages. The open approach is simple and reliable for surgeons of all levels of experience but requires a long skin incision and is more invasive than the other two approaches. Additionally, there is some possibility of complications, such as stiffness and minor wound healing issues. The endoscopic approach is performed using an ankle hindfoot endoscope^10^ for checkrein deformities. Keeling and Guyton^11^ reported on an endoscopic FHL decompression procedure in cadavers; they observed that the endoscopic technique is reliable and less invasive than the open technique but that it is technically demanding, with significant risk to local neurovascular structures. In our view, endoscopic FHL tenotomy is simpler than endoscopic FHL decompression, but the advantages and disadvantages of these procedures are almost the same (Table 2). In terms of the present technique for FHL tenotomy under ultrasound, surgeons have to be familiar with posteromedial ankle anatomy and must be well experienced using ultrasound. However, this procedure requires just 1 skin incision (the hindfoot endoscope procedure requires 2 skin incisions), and the FHL tendon can be accessed via the shortest route because the FHL tendon itself can be detected by ultrasonography. Additionally, the position of the NVB can be seen under ultrasonography, so it can be safely protected. Furthermore, if FDL lengthening is required in a patient, it can be performed at the same position. In conclusion, ultrasonography-guided FHL tenotomy for the checkrein deformity of the hallux requires ultrasound training, but this procedure is very promising because it is safe and less invasive than other procedures.

**Table 2:** Advantages and Risks and/or Limitations of Ultrasonography-Guided Flexor Hallucis Longus Tenotomy

Advantages
Low invasiveness
Cosmetically superior with small skin incisions
Ability to walk on day 1 after surgery
No need for external fixation after surgery
Ultrasound can be used to reliably prevent neurovascular damage
Risks and/or limitations
Surgery under ultrasonography requires long learning curve
Ultrasound system with high resolution is needed
May decrease flexion power of great toe

## Disclosures

The authors declare the following financial interests/personal relationships which may be considered as potential competing interests: A.N. receives speaking and lecture fees from Arthrex, United States. All other authors (S.N., Y.S., N.I., A.F., K.K., M.H.) declare that they have no known competing financial interests or personal relationships that could have appeared to influence the work reported in this paper.

## Supplementary Data

Video 1 Step-by-step demonstration of ultrasonography-guided flexor hallucis longus tenotomy of the left foot. The surgical setup, anatomic identification with ultrasound, lidocaine injection, blunt dissection, tendon isolation, and tenotomy are shown. Each phase is accompanied by practical tips for safe performance.
